# Supplementary figures and images for: Brucella suppress STING expression via miR-24 to enhance infection
Source: PLoS Pathog. 2020 Oct 27;16(10):e1009020. doi: 10.1371/journal.ppat.1009020 (PMC7647118; doi:10.1371/journal.ppat.1009020)

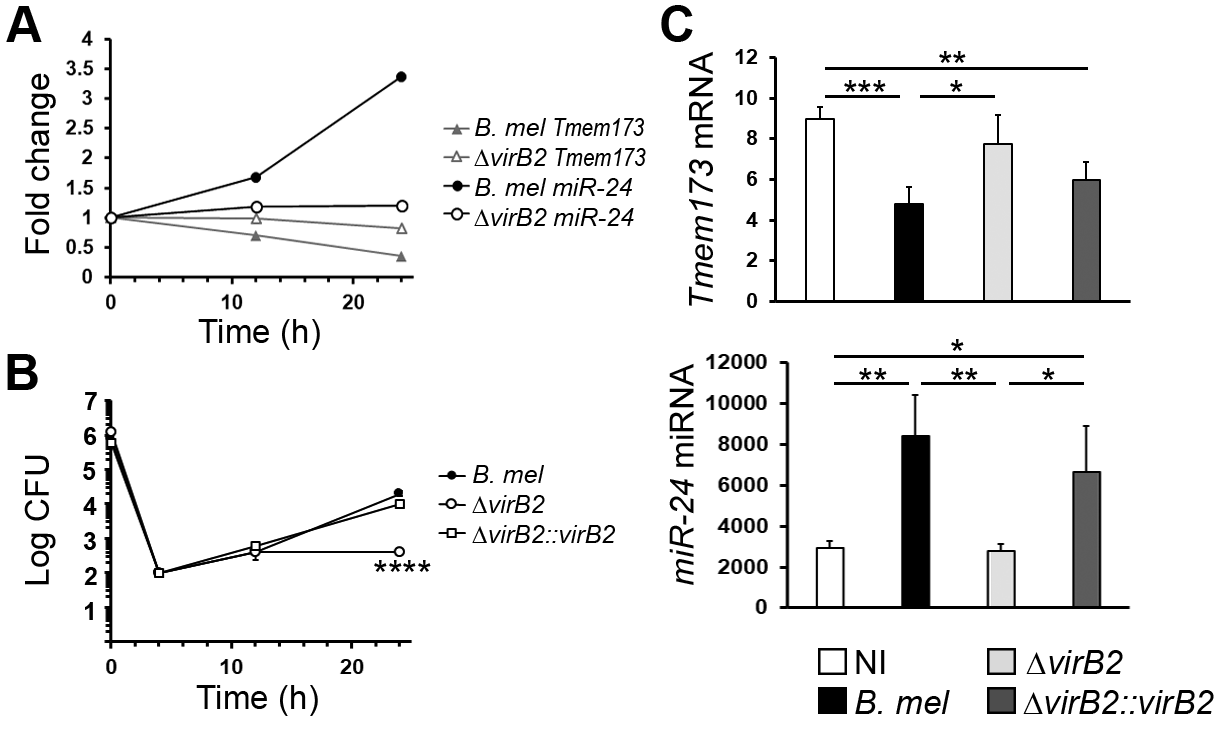

Supplement: S1 Fig — A) Bone marrow derived macrophages were infected with 100 MOI B. melitensis or ΔvirB2 mutant and harvested for RNA at 12h and 24h post infection. Tmem173 and miR-24 expression was normalized to Time 0 for each genotype (set = 1). B) Immortalized macrophages were infected as above and CFU (8 replicates) determined over time. By 24h, the ΔvirB2 mutant was at a significant disadvantage for replication (p<0.001) vs. wild type B. mel and the complemented mutant. C) Macrophages were infected with 100 MOI of B. melitensis, ΔvirB2 mutant, or the mutant transfected with the virB2 gene for 8h prior to harvest for RNA. Error bars are triplicate standard deviations. (TIF) [file ppat.1009020.s001.tif]

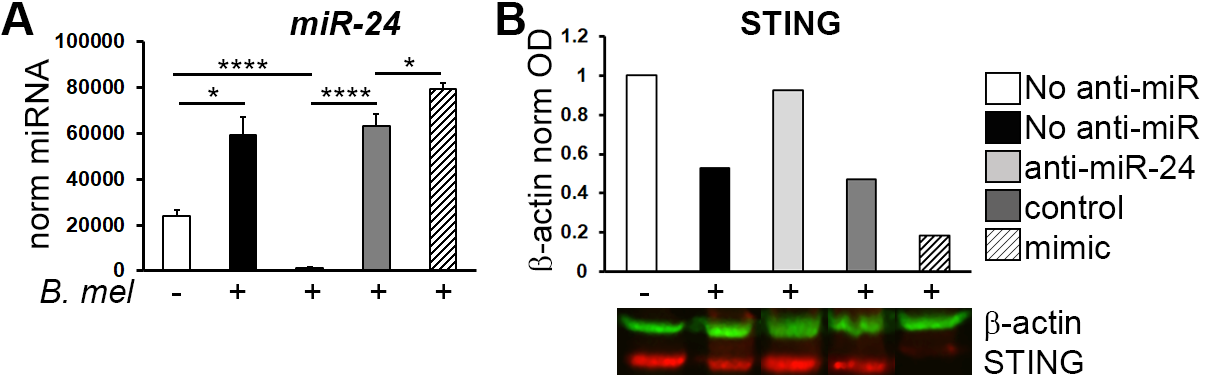

Supplement: S2 Fig — A) Macrophages were not transfected (white and black bars) or transfected with anti-miR-24 inhibitor (pale gray), scrambled control nucleotide (dark gray), or miR-24 mimic (striped bars). Cells were subsequently uninfected (-) or infected with 100 MOI B. melitensis (+). After 24h, cells were harvested for RNA and miR-24 levels determined by qPCR with normalization to RNU6. N = 3 experiments. Error bars are SEM. *p<0.05, ****p<0.001. B) Macrophages were transfected as in (A). 24h following infection with Brucella, cells were lysed and whole cell lysates resolved by SDS-PAGE. STING and β-actin were detected using western blot and immunofluorescence. Blot is representative of 2 experiments. Graph bars are actin-normalized optical densities of the STING bands. (TIF) [file ppat.1009020.s002.tif]

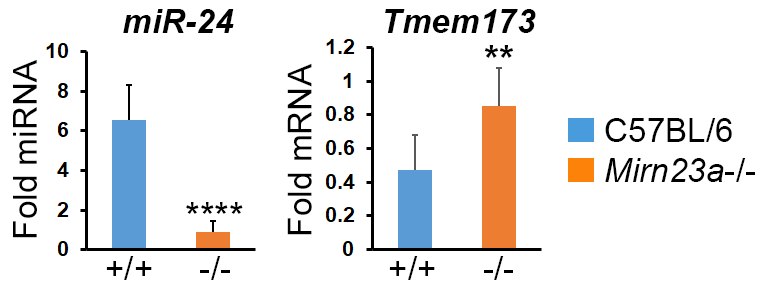

Supplement: S3 Fig — C57BL/6 (+/+) and Mirn23a-/- (-/-) mice were infected intra-peritoneally with 106 B. neotomae (7 mice per group). After 7 days, mice were sacrificed and spleens harvested for CFU (Fig 7) and RNA. Gene expression was determined by qPCR. Results from individual infected mice were normalized to an uninfected control (set = 1) for each genotype. ****p<0.001 and **p<0.01 vs. infected C57BL/6. (TIF) [file ppat.1009020.s003.tif]
