# Supplementary material for: Brucella suppress STING expression via miR-24 to enhance infection
Source: PLoS Pathog. 2020 Oct 27;16(10):e1009020. doi: 10.1371/journal.ppat.1009020 (PMC7647118; doi:10.1371/journal.ppat.1009020)
Supplement: Attachment — Submitted filename: Author response073020.docx [file ppat.1009020.s004.docx]

July 30th, 2020

To: Plos Pathogens editorial office

RE: revision of PPATHOGENS-D-20-00042

Dear Editors and Reviewers,

I am writing to resubmit our manuscript originally entitled “*Brucella* suppresses innate immunity by down-regulating STING expression in macrophages”. Thank you for the opportunity to respond to the critiques! Thank you to the reviewers for your careful reading of the manuscript and attention to detail. We have endeavored to respond fully to all the critiques and our point- by-point response is below. We think the new experiments have significantly strengthened the manuscript and hope the reviewers agree. Reviewer comments are italicized.

Part I: -- Summary

*1. The major concern is the novelty of the data presented in this manuscript. It has been well established that miR-24 represses Tmem173 expression (PMID: 22387590 and 31735332). Furthermore, the role of STING in limiting Brucella infection has also been previously demonstrated. The innovative part of this manuscript is the active upregulation of miR-24 by Brucella which requires a functional T4SS*.

We felt it was important to start the paper with a confirmation of previous results to indicate why *Brucella* would benefit from suppressing STING expression, “setting the stage for the story”. Regarding the previous miR-24 literature: PMID 22387590 is a report from Developmental and Comparative Immunology focused on cloning of the rat STING gene that briefly described its post-transcriptional suppression by miR-24. PMID 31735332 came out after our manuscript was first submitted and highlights the role of miR-24 in suppressing STING in the setting of liver ischemia-reperfusion injury. We were happy to add this reference to the manuscript. The suppression of STING, via miR-24 during intracellular bacterial pathogen infection is highly novel. Additionally, it is very unusual for a single miRNA to have this much impact on infection.

2. *Adding more dating to this manuscript to investigate the mechanism of Brucella-induced upregulation of miR-24 (possibly mediated by an effector protein, or intracellular PAMPs (e.g. LPS) independent of MyD88 signaling) would greatly enhance this manuscript.*

As requested, we have examined the effect of other previously characterized *Brucella* PAMPs in addition to the investigation of MyD88 and STING, including purified TLR2, TLR9 and TLR4 agonists. *Brucella abortus* and *melitensis* have “smooth” LPS, which is a much poorer TLR4 stimulus than LPS from most Gram- bacteria (e.g. *E. coli*). Therefore, we took the additional step of purifying *Brucella* LPS. None of these PAMPs suppressed STING expression (revised Figure 5).

*3. Furthermore, all experiments (except Figure 1B) are performed with immortalized BMDMs and in vivo evidence (mouse infections) is lacking showing that Brucella upregulates miR-24, resulting in STING suppression and increased bacterial numbers. Mirn23a-/- mice should be available to the authors, since mirn23a-/- macrophages were used in this study. What is the effect of mirn23a deficiency on Brucella infections (chronic and/or acute) in vivo?*

We added an experiment in primary BMDM repeating the major findings regarding miR-24, *Tmem173* and VirB (Figure S1). We added *in vivo* evidence for miR-24 upregulation (Figure 4). *Mirn23a-/-* bones were much easier to obtain than mice. The COVID shut down of labs and vivaria at both UW-Madison and U of Indiana (where the *Mirn23a-/-* colony is maintained) complicated matters. After getting the safety regulatory paperwork done, our collaborators were able to obtain just enough mice and had just enough time to do a big one-week experiment. Fortunately, the outcome *in vivo* perfectly paralleled what we observed *in vitro* for replication: the *Mirn23a*-/- mice had lower CFU, suggesting this locus supports infection *in vivo* (revised Figure 7).

*Minor comments:
4. Figure 3B. An intact T4SS is required for Tmem173 expression, but does deletion of the T4SS result in lower numbers of intracellular bacteria that could account for the partial rescue of Tmem173 expression? Possibly the amount of intracellular bacteria, or PAMPs, could lead to miR-24 upregulation*?

To address this issue, we examined the kinetics of *Tmem173* and miR-24 changes with the VirB mutants and CFU. *B. melitensis* seems to be less dependent upon the T4SS than what has previously been published using *B. abortus*. Our *in vitro* findings would go along with *in vivo* data described by Splitter’s group (PMID:16153245) whereby VirB was required for persistence in mice, but the first 3 days growth were similar to wild type. We found that the Δ*virB2* *B. melitensis* mutant CFU did not begin to diverge from wild type in macrophages until after 12h, well past the point when differences in host gene expression were evident. There is some modest suppression of *Tmem173* in the VirB mutants, which occurs before the type IV secretion system is fully induced (~4h post-infection). This initial suppression may indeed be due to PAMPs besides TLR/STING agonists or another unidentified factor. The VirB mutants are reliably unable to upregulate miR-24, supporting the idea that a small amount of STING mRNA suppression occurs independently of both T4SS and miR-24.

*5. The authors should rephrase sentence 162/163. Statistically there was no significant increase of miR-24 in Brucella infected MyD88-/- macrophages compared to uninfected MyD88-/- macrophages, suggesting MyD88 might be required in miR-24 expression (even though there was still a small increase in miR-24 expression in MyD88-/- macrophages).*

We had rounded the p-value on the figure: the original p-value was 0.065. This p-value does not fully represent the result, since the variability was increased by normalizing each data point to the wild-type non-infected sample within each experiment. This was originally done to determine if there were any baseline gene expression differences in non-infected macrophage genotypes. To address your point, a panel has been added in which MyD88 NI was set=1 within each experiment. As is now evident, upregulation is statistically significant (p<0.005).

**Part II – Major Issues: Key Experiments Required for Acceptance**
*6. Reviewer #1: As outlined above (Part I), the authors should 1) investigate the mechanism of Brucella-induced expression of miR-24 and 2) provide evidence of the in vivo (mouse experiments) relevance of miR-24 expression during Brucella infections.*

We have performed the additional PAMP experiment and consolidated the importance of the T4SS. Any further investigation of the mechanism is outside the scope of this initial report. *In vivo* experiments have been added. See point 3 for further details.

*7. Reviewer #2: 1) Regarding the results show in Figure 1 respect to the role of STING in the control of Brucella infection in vivo and in vitro. Results seem to be mentioned as complementary of previous studies but to really know if the control of infection begins at early time points (as it seems to this reviewer, and is suggested by the authors in the discussion section, lines 273-274) or late in the chronic phase of infection; as the experiments performed in vivo in this Figure and stated in the title of Results. Thus, a kinetic should be performed in vitro (12, 24, 72 h) and in vivo (1, 3 and 6 wk) under the same experimental conditions to understand how early or late STING is controlling Brucella infection*.

24, 48 and 72 hours *in vitro* have been previously published (Costa Franco 2018, now referenced). We felt it was important to show an additional, confirmatory repeat of the 24h effect, since so many of the experiments were performed at this time point. Per request, our collaborator with the STING-/- mice has sent an additional *in vivo* experiment containing all three time points (Figure 1B).

*8. Scramble control of transfection with anti Mir-24 used to show specificity does not work properly. Levels of mir24 expression should be comparable to the ones achieved with infection in the absence of specific mir-24 inhibition, thus invalidating the results and conclusions of Fig1 s and more important results in Figure 6. Please perform again the experiments (Fig1 S and Fig 6) using another scramble control that allows clear conclusions on the results obtained*.

There was an issue with detection and how the data from the initial experiments for Figure S1 were normalized. We repeated the assay and are now showing raw qPCR data (only normalized to RNU6 housekeeping RNA) for miR-24, which should make a lot more sense given what we observed with STING mRNA and protein.

*9. Although the use of v-raf/v105 myc immortalized murine bone marrow-derived macrophages is convenient and reliable, at least in when they need to be transfected. Some experiments would favor if primary BMDM are use. For instance, downregulation of STING expression due to Brucella species and the requirement of live bacteria and TSS4 system should be performed in WT BMDM to demonstrate the universality of the phenomenon.*

This has been done (Figure S1) and reflects the same results obtained with the immortalized cells. In our experience, these immortalized BMDM have proven much more faithful in recapitulating results from primary BMDM than any other macrophage cell line we have tried.

*10. Reviewer #3: I really only have one concern. In my opinion, with the experiments that were carried out authors cannot conclude the phenotypes are dependent on the T4SS. At 24h, the virB mutant is highly attenuated so there is 1) a significant difference in number of bacteria in cells infected between the wt and mutant and 2) the mutant is in a completely different intracellular compartment. Both of these could indirectly impact the results and give the false impression that the T4SS is necessary.

Authors could try to do these experiments at earlier time-points were trafficking and CFU counts are comparable between the WT and the virB mutant or simply remove these data, which are not essential for any of the conclusions presented.*

 See point 4 above describing the inclusion of gene expression kinetic data (Figure 4G), an earlier time point in BMDM and CFU (both in Figure S1). We hope you are convinced that gene expression divergence from wild type occurs well before CFU attenuation. We also provided a complementation assay at 8h post-infection (Figure S1)

**Part III – Minor Issues: Editorial and Data Presentation Modifications**

*11. Reviewer #2: 1) Title does not reflect the results obtained in the investigation. It should refer to the role of mir24 in STING down-modulation. Indicating the role of STING in innate immunity is an overstatement. Also the role of STING in innate immune responses has been already investigated and published by Costa Franco MM et al (J Immunol. 2018 ;200(2):607)*

The title has been changed to “*Brucella* suppress STING expression via miR-24 to enhance infection”

12*. Figure 2 A: why STING expression was evaluated a number of very different times depending upon the species investigated? Melitensis 25 times, Abortus 7 and the other 2 species 5 times ….Results does not seem to be variable? Please explain*.

There were only 5 experiments done to compare STING suppression vs. non-infected cells using the 4 different *Brucella* species. *B. abortus* was used in at least 2 additional experiments in comparison with *B. melitensis*. *B. melitensis* was used for almost all other experiments in the manuscript. In Figure 2, we wanted to include as much data as we could to support the observation of STING suppression by the different *Brucella* species. Interestingly, all 4 species showed evidence for similar degrees of STING suppression. These corroborating data were important to establish for experiments done in collaborating labs where we could only use *B. abortus* or *B. neotomae*.

*13. Figure 2B: please show a more representative WB that the one depicted. Beta-actin seems to vary in the different lanes. Since the experiment was repeated several times as referred in the Figure legend a more representative photo must be shown. Why the picture has 3 lanes when the bar graph only has 2?*

In that experiment, there was an extra well of infected cells. We have provided another experiment in which the degree of protein suppression is representative of the bar graph depicted average.

*14. Figure 3 C: It is not clear what the asterisks in the HK bar stand for? In the texts is stated that HK is “comparable “to WT (line 120). Please clarify*

P-values are infected vs. NI (non-infected). One experiment showed a 4-fold induction of luciferase by live *Brucella*, increasing the variability and thus diminishing the p-value, but all experiments had at least a 50% (1.5-fold) increase in activity. This is now clarified in the legend and stated explicitly in the results text.

*15. Overall figure legends are no easy to follow. Modify them to make them fit with what is shown in the figures*

As requested, we have made an effort to clarify the legends.

*16. Figure 4 A: Why if in the text (line 147) states that mir-24 levels vary between 50% and 8 times, the dispersion shown in the figure is so small (4 +/- 1 times). What kind of replicate were included in the data?.*

The experiment containing an uninfected sample and *Brucella*-infected sample was performed at least 17 independent times (!), as indicated in the Figure legend. Most of the experiments were done with at least duplicate samples, and averages from individual experiments used to generate the data for the graphs. These are all independent experiments, so the appropriate error bar is the standard error of the mean. SEMs are smaller than standard deviations because they are adjusted for the number of times the experiments were performed.

*17. Discussion: Lines 304-305 are an overstatement to say that “Evades full activation of innate immunity”. It should say modifies IFN beta expression and apoptosis at the most*

This has been revised to “evades full activation of STING by downregulating its expression via miR-24.” Although we examined Bim mRNA, we cannot address apoptosis at this point.
